# High quality self-separated GaN crystal grown on a novel nanoporous template by HVPE

**DOI:** 10.1038/s41598-018-21607-3

**Published:** 2018-02-16

**Authors:** Qin Huo, Yongliang Shao, Yongzhong Wu, Baoguo Zhang, Haixiao Hu, Xiaopeng Hao

**Affiliations:** State Key Lab of Crystal Materials, Shandong University, Jinan, 250100 P.R. China

## Abstract

In this study, a novel nanoporous template was obtained by a two-step etching process from MOCVD-GaN/Al_2_O_3_ (MGA) with electrochemical etching sequentially followed by chemical wet etching. The twice-etched MOCVD-GaN/Al_2_O_3_ (TEMGA) templates were utilized to grow GaN crystals by hydride vapor phase epitaxy (HVPE) method. The GaN crystals were separated spontaneously from the TEMGA template with the assistance of voids formed by the etched nanopores. Several techniques were utilized to characterize the quality of the free-standing GaN crystals obtained from the TEMGA template. Results showed that the quality of the as-obtained GaN crystals was improved obviously compared with those grown on the MGA. This convenient technique can be applied to grow high-quality free-standing GaN crystals.

## Introduction

Gallium nitride (GaN) is an important wide band gap semiconductor that has been applied widely in the optoelectronic^1,2^ and high-power high-frequency devices^3^ because of its excellent optoelectronic and electronic properties. However, almost all of the GaN-based devices are hetero-epitaxially grown on foreign substrates due to the lack of GaN substrates for homo-epitaxy. The performances of these devices are greatly decreased because of the high threading dislocation density caused by the large lattice and thermal expansion coefficient mismatches between the foreign substrates and GaN epitaxial layer^4^.

The hydride vapor phase epitaxy (HVPE) method is recognized as the most successful and promising method to grow large-sized bulk GaN crystals^5^. The advantages of HVPE include simple equipment, low cost and quick growth speed. However, the mismatch issues trouble the HVPE growth of the GaN on foreign substrates. To reduce residual stress and dislocation density caused by the mismatch issues in the HVPE process and fabricate freestanding GaN, several kinds of substrates have been designed. The epitaxial lateral overgrowth (ELOG) processes on various patterned substrates have been widely used to reduce the defect density and strain^6,7^. Oshima *et al*. fabricated freestanding GaN substrate by using the void-assisted separation (VAS) method^8^. In the VAS method, a GaN/sapphire template covered with a TiN nano-net was used as the base substrate^9^. NH_4_Cl^10^ and SiN_x_^11^ were also reported to be covered on substrates as a release layer to fabricate freestanding GaN. However, all these substrates are too inconvenient or expensive to be fabricated. Etching the GaN/sapphire templates is a frequently used way to fabricate the buffer layer. The wet chemical etching^12^, *in situ* etching^13^, electrochemical etching^14^ and the reactive ion etching (RIE)^15^ methods have been reported. Nanomaterials have been also applied in the HVPE process of GaN growth. Moonsang Lee *et al*. grew thick GaN via GaN nanodot formed by HVPE^16^. Other nanomaterials, including graphene or hexagonal boron nitride nanosheets^17^ and cross-stacked carbon nanotubes^18^, have been employed also as a release structure for GaN growth. These nanomaterials have efficiently reduced the stress of GaN crystals, but the manual coating procedure needs too many skills so that the fraction of usable substrates is relatively low.

To reduce the residual stress and realize the self-separation of GaN crystals, our group has explored several methods, including wet chemical etching^19^, high temperature annealing^20,21^ and electrochemical etching methods^22^, to fabricate the nanoporous GaN buffer layer. However, the wet chemical etching method and the high temperature annealing method cannot be well controlled because of the anisotropic chemical activity of GaN crystal^23^. Meanwhile, it is difficult to obtain high-porosity and large-sized nanoporous structure which is important for the self-separation of GaN crystals by the electrochemical etching method^24^.

In this paper, we designed a simple two-step etching technology by combining the advantages of wet chemical etching and electrochemical etching based on the different chemical activity of N-polar and Ga-polar GaN. In this method, the surface morphology and the porosity of the etched substrate can be regulated independently. The substrate with relatively uniform diameter and density of etching pits can be obtained in electrochemical etching. By enlarging the porosity of the substrate through N-polar wet chemical etching, the TEMGA template was fabricated. Because of the mild condition of the electrochemical etching and low temperature N-polar wet chemical etching, this process can be well controlled and the substrates are easily reproduced. The much higher porosity of TEMGA template is more efficient to the self-separation of HVPE grown GaN crystals than that of electrochemical etching substrates. The quality of GaN crystals grown by HVPE on TEMGA template and MOCVD-GaN/Al_2_O_3_ (MGA) template was researched to indicate the effect of the two-step etching process. Acquiring free-standing GaN crystals with high crystalline quality utilizing the novel developed porous template will be convenient.

## Experimental

An MGA template with a GaN layer of about 5 μm in thickness, fabricated by MOCVD on a 2-inch c-plane sapphire substrate, was employed in the subsequent experiments. The upper Si-doped n-GaN (n = 8 × 10^18^ cm^−3^) layer is about 3 μm thick and the lower undoped GaN layer is about 2 μm. At first the template with n-GaN were etched by electrochemical method. 0.3 M oxalic acid was used as the electrolyte, n-GaN as the anode and Pt electrode as the cathode. The voltage of 15 V was applied for the electrochemical system for 10 min to obtain the suitable nanoporous structure. Then, the nanoporous GaN was etched in H_3_PO_4_ at 90 °C for 2 h to enlarge the nanoporous of N-polar face at the bottom of n-GaN layer. The TEMGA template was obtained by the twice-etched process. The TEMGA template was washed with the deionized water and dried by N_2_ gas. The dried template was used as the substrate in the next growth process. After cleaning, the TEMGA template was loaded as the substrates in a home-made vertical HVPE reactor for GaN crystal growing. The growth was performed at atmospheric pressure. Liquid Ga and NH_3_ gas were used as Ga source and N source, respectively. N_2_ gas was used as the carrier gas. The temperature of the Ga-boat where HCl gas and liquid Ga formed GaCl was 850 °C, and the temperature of the substrate where GaCl and NH_3_ formed GaN was 1050 °C. The growth rate of the HVPE layer was about 100 μm/h. After 12 h of growth process, the 1.2-mm thick GaN crystal was obtained. The morphology of the twice-etched nanoporous structure was observed by field emission scanning electron microscopy (FE-SEM, Hitachi S4800). The diameter and density of the etching pits were calculated by the software (ImageJ v1.80) from the SEM images. Raman spectra of the HVPE-GaN crystals were obtained by the LabRAM HR system of Horiba Jobin Yvon at room temperature using a 532 nm solid laser as the exciting source. Photoluminescence (PL) measurements were carried out at room temperature using 325 nm He–Cd lasers as the excitation source. The quality of the HVPE-GaN crystals grown on different templates was characterized by high-resolution X-ray diffraction (HRXRD, PANalytical X’PERT PRO). An EBSD system (Oxford Instruments INCA Crystal EBSD system, Nordlys EBSD Detector, and HKL CHANNEL5 software) mounted in the FE-SEM was used to characterize the HVPE-GaN crystal orientation. The EBSD measurements were carried out at 20 kV with a working distance of 20 mm and a sample tilt of 70°.

## Result and Discussion

**Figure 1:**
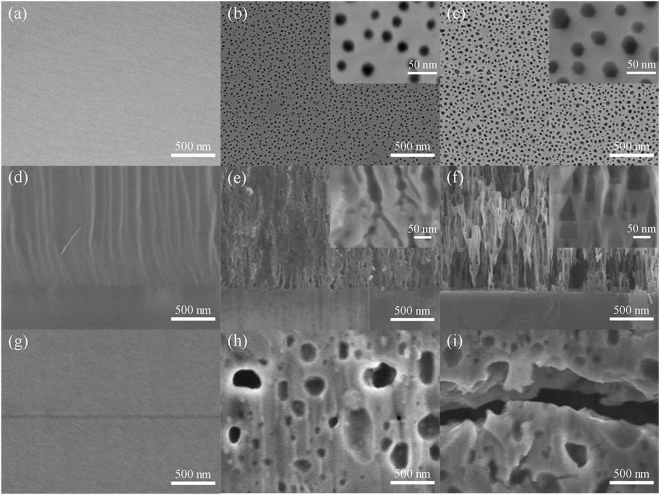
SEM images of the top view (**a**) and cross-section view (**d**) of MGA template. SEM images of the top view (**b**) and cross-section view (**e**) of the porous structure formed by the electrochemical etching. SEM images of the top view (**c**) and cross-section view (**f**) of the porous structure formed by the twice-etching. SEM images of the cross-section view of the GaN crystals grown on MGA template (**g**), ECMGA template (**h**) and TEMGA template (**i**), respectively.

The nanoporous structure of substrate is important for the reduction of stress and dislocation in HVPE-GaN crystal^25^. Furthermore, the HVPE-GaN growth mode adjustment and its self-separating process from TEMGA template are influenced by the morphology of the nanopores. To identify the structure of twice-etched nanopores, the morphology of the nanoporous template in each etching step was researched. The morphology of the MGA template is also shown as a control group. The top view (Fig. 1(a)) and the cross-section view (Fig. 1(d)) of the MGA template shows that no nanopores appeared after etching. The top view of the nanoporous structure prepared by electrochemical etching is shown in Fig. 1(b). It can be seen that most of the etching pits were round holes. The average diameter of the etching pits was 20 nm and the density of the etching pit was 4 × 10^10^ cm^−2^. The branch porous structure can be seen clearly in the cross-section of the electrochemical etched nanoporous structure (Fig. 1(e)). As reported, the scale, shape and density of the nanopores were controlled by both the bias voltage and the doper concentration^26^. The weight loss of the TEMGA template was 27% of the Si-doped GaN layer. This result agrees with the estimated 30% porosity, as shown in Fig. 1(e).

To obtain high-porosity and large-sized nanoporous structure which is important for the self-separation of GaN crystals, the as-obtained electrochemical etching template was subsequently etched by wet chemical method. Figure 1(c) shows the top view morphology of the nanoporous structure formed by wet chemical etching in H_3_PO_4_ for 2 h at 90 °C after the electrochemical etching process. From these results, we found that the density of the etching pits after H_3_PO_4_ etching maintained at 4 × 10^10^ cm^−2^. However, the size and shape changed. The scale of etching pits on the TEMGA template was enlarged from about 20 nm to 30 nm, and the shape of etching pits was transformed from round to hexagon. This observation indicated that few new etching pits emerged through H_3_PO_4_ etching. It also suggested the chemical inert of the Ga-polar GaN surface. Figure 1(f) shows the cross-section morphology of twice-etched nanoporous structure. The layer with inverted pyramid porous structure can be seen clearly. The unique invert triangles of the inverted pyramid porous structures in the TEMGA template were found not only at interface but also in the interior of the Si-doped GaN layer. This cross-section morphology was caused by the chemical activity difference between Ga-polar and N-polar GaN. In the previous research, the difference of the chemical property between Ga-polar and N-polar GaN surface was reported^27^. N-polar GaN surface was etched in H_3_PO_4_ at 90 °C in some reports^28^. In contrast to our previous research, Ga-polar surface had no change in H_3_PO_4_ even at 210 °C^23^. Meanwhile, the etching rate of N-polar was much faster than that of Ga-polar^29^. When the nanoporous-GaN was etched at this relatively low temperature, Ga-polar surface of GaN did not react with H_3_PO_4_ thereby showing minor change. However, N-polar face of GaN could be etched in H_3_PO_4_. N-polar GaN was exposed not only at the interface but also at the treetop in the branch porous structures. H_3_PO_4_ can arrived the treetop of the branch porous structures through these pores. Then, the wet chemical etching reaction started not only at the interface but also at all of the tips of the branch pores. Then, the hierarchy inverted pyramid porous structures shown in Fig. 1(f) were formed. The weight loss of the TEMGA template was 45% of the Si-doped GaN layer. This result agrees with the estimated 49% porosity, as shown in Fig. 1(f). After the H_3_PO_4_ etching, the inverted pyramid porous structures were formed and the porosity of the nanoporous GaN has been greatly increased. By this method, we obtained the high-porosity nanoporous substrates without heavily damaged surface.

The TEMGA template was employed for HVPE GaN crystal growth. The growth mechanism of GaN on the nanoporous structure is important for the reduction of residual stress and self-separation process. To identify the effect of the nanoporous structure on HVPE-GaN growth, the morphology of GaN on TEMGA template was researched. Figure 1(g–i) show the cross-section morphology of the HVPE GaN grown on the TEMGA template, electrochemical etching MOCVD-GaN/Al_2_O_3_ (ECMGA) template and MGA template, respectively. It is observed that there were many voids at the buffer layer of the HVPE-GaN crystals both grown on TEMGA and ECMGA templates. Meanwhile, no void was observed at the buffer layer between the HVPE-GaN and the MGA template. This finding means that the voids at the buffer layer after the HVPE-GaN growth originated from the branch porous structure and the inverted pyramid porous structure. At the same time of the high temperature overgrowth, the pores arched through the surface and gas phase diffusion were driven by the surface energy. This deformation could be explained through Rayleigh instabilities, in which atoms diffuse from the larger curvature region toward the lower curvature region of the cylinder surface to reduce the surface energy^30^. The initial shape of the nanopores determines the final shape of nanopores after deformation. After HVPE growth, the branch porous structures formed by electrochemical etching were transformed into small and isolated voids, whereas the inverted pyramid porous structures formed by two-step etching were transformed into well-aligned voids^14^. The distinction of the deformation morphology comes from the difference of porosity and pore size. The well-aligned voids are essential to the self-separation between the regrown HVPE-GaN from the substrate.

**Figure 2:**
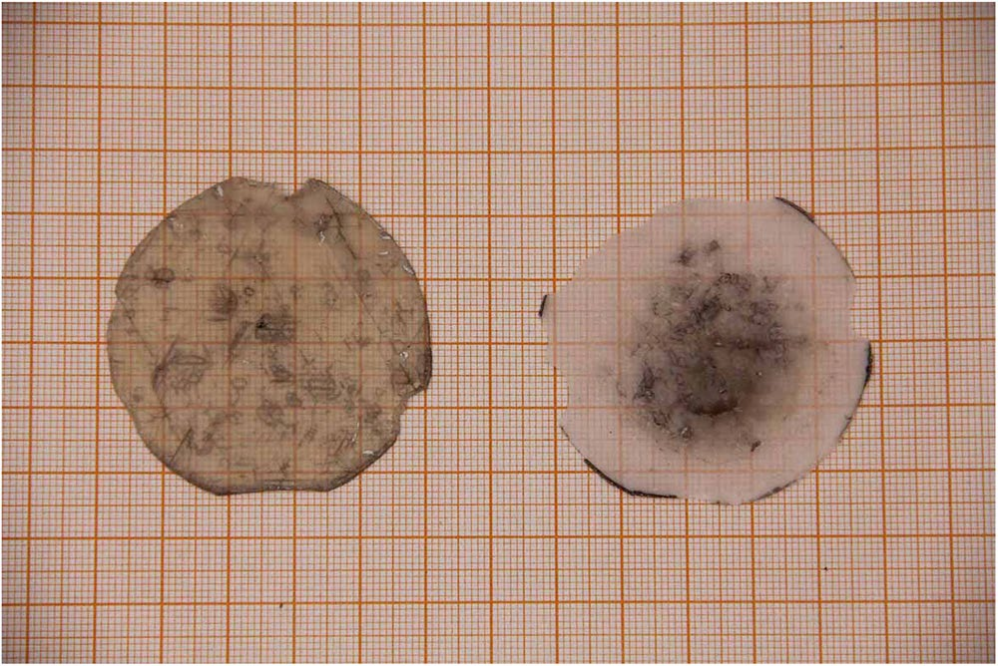
Photos of the 2-inch lift-off GaN crystal grown on the TEMGA template (left) and the residual TEMGA template (right).

The photos of the 2-inch lift-off GaN crystal grown on the TEMGA template (left) and the residual TEMGA template (right) are shown in Fig. 2. The entire 2-inch HVPE-GaN crystal is separated from the substrate. The thickness of the 2-inch lift-off GaN crystal wafer is 1.2 mm. There are still some dark spots and cracks in the free-standing GaN crystal wafer. The dark spots are on the top of the GaN crystal and the cracks are almost at the bottom of the GaN crystal. The dark spots on the crystal are caused by the deterioration during the GaN crystal growth. With the crystal growth, parasitic polycrystals are deposited on the air gate of the GaCl and NH_3_. On the later stage of the GaN crystal growth, dark spots emerged because the fluent of the gas is interfered by the parasitic polycrystals. And the cracks are caused by the thermal stress at the interface between substrate and GaN crystal during the cooling process. Although the buffer layer can release the stain and reduce the cracks of the GaN crystal, the self-separation may crack the crystal during the cooling process due to the high cooling rate and large thermal stress. Further growth optimization needs to be carried out to eliminate the dark spots and cracks.

**Figure 3:**
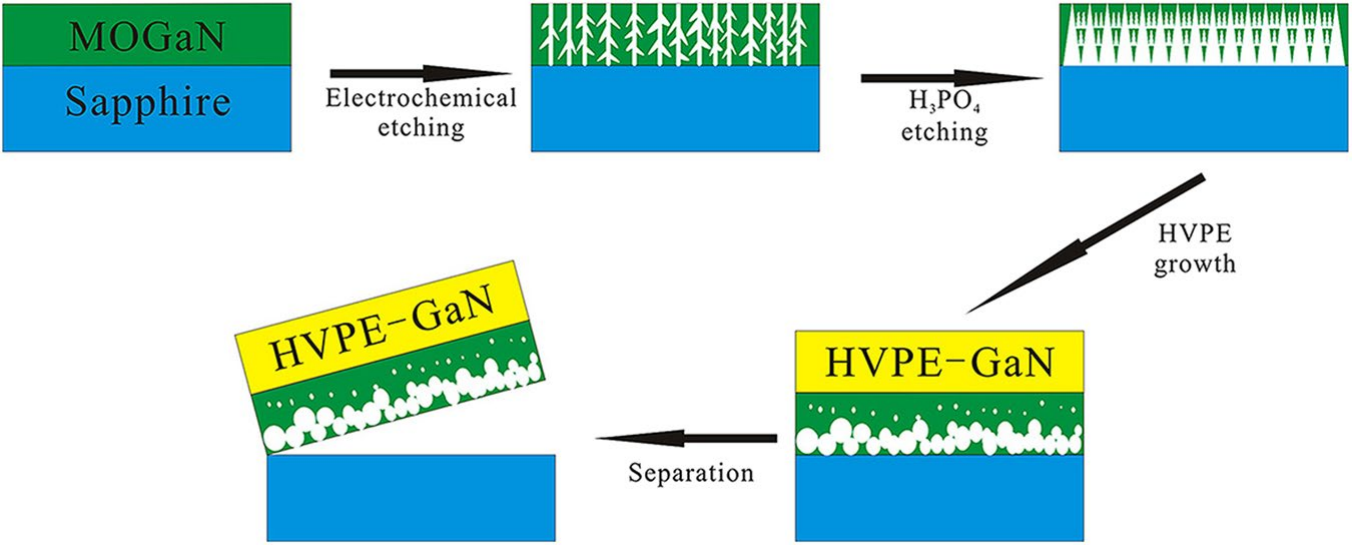
Schematic diagram of the self-separating GaN crystal grown on the TEMGA template.

The process and mechanism of HVPE-GaN crystals self-separating from the TEMGA template are depicted in Fig. 3. The nanoporous structure is formed by electrochemical etching process, and then enlarged by the wet chemical etching process. The nanoporous structures are transformed into voids in the buffer layer during the crystals growth process. Because of the mismatch in thermal expansion coefficient between substrate and GaN crystal, strong stress occurs at the interface between substrate and GaN crystal in the cooling process. And then the weak links between the GaN crystal and substrate in the buffer layer are broken. Finally, the GaN crystal self-separates spontaneously from the TEMGA template.

**Figure 4:**
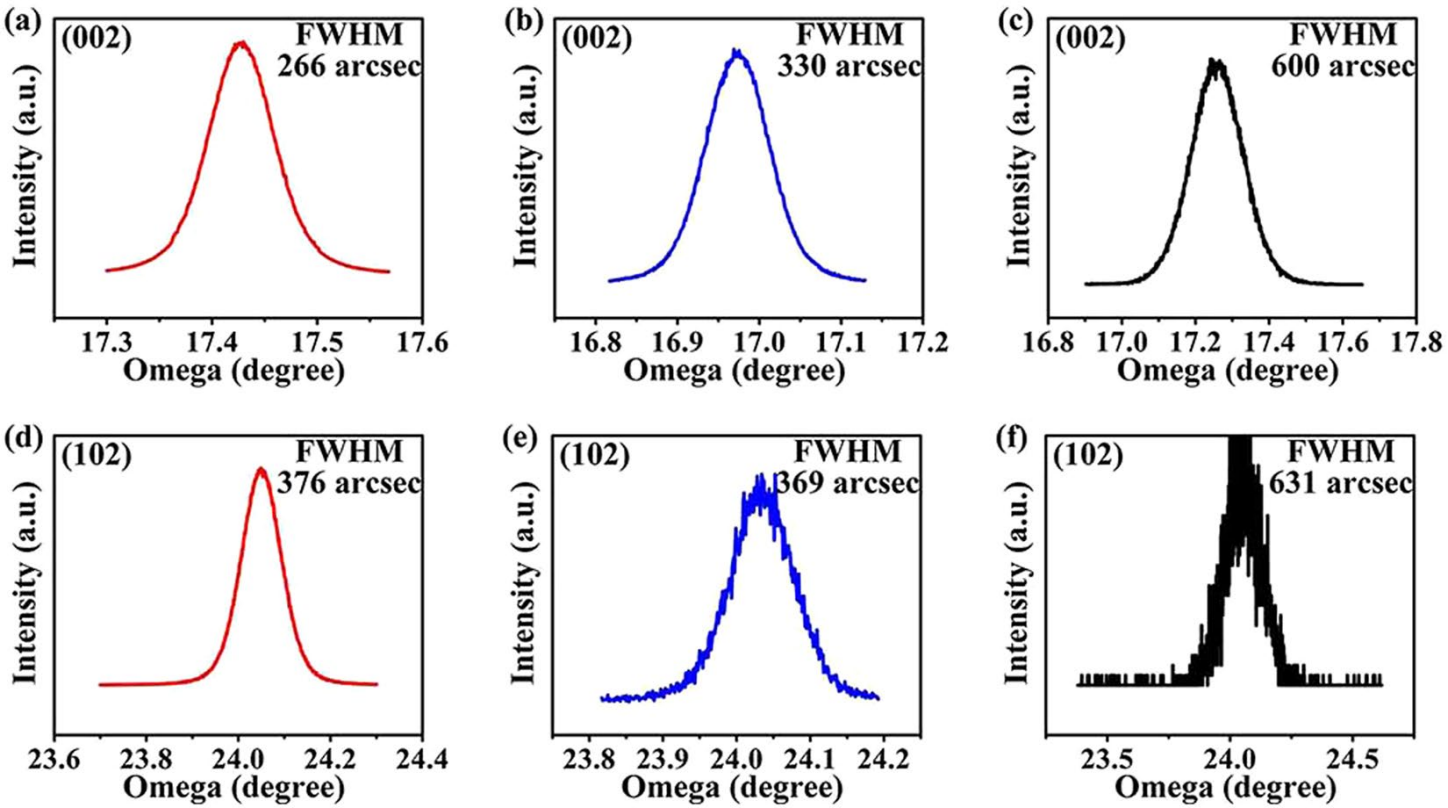
The ω-scans HRXRD rocking curves of the HVPE GaN crystals grown on TEMGA ((002) symmetrical peak (**a**) and (102) asymmetrical peak (**d**)), ECMGA ((002) symmetrical peak (**b**) and (102) asymmetrical peak (**e**)) and MGA ((002) symmetrical peak (**c**) and (102) asymmetrical peak (**f**)) substrate.

To research the effect of nanoporous structure formed by twice-etching on the HVPE grown GaN crystal, the crystalline quality and stress condition were identified. The crystalline quality of the HVPE-GaN was characterized by a high-resolution X-ray diffraction (HRXRD) rocking curve. Figure 4 depicts the ω-scan of the (002) symmetry planes and the (102) asymmetry planes of the HVPE-GaN crystals grown on the different substrates. The full width at half maximum (FWHM) of the (002) peak are 226 arcsec, 330 arcsec and 600 arcsec for HVPE-GaN crystals that grown on the TEMGA, ECMGA and MGA templates, respectively. And the FWHM of the (102) peak are 376 arcsec, 369 arcsec and 631 arcsec for HVPE-GaN crystals that grown on the TEMGA, ECMGA and MGA templates, respectively. It is well known that the FWHM of the HRXRD rocking curve for the symmetrical (002) plane is related to screw and mixed dislocations, and the FWHM of the HRXRD rocking curve for the asymmetrical (102) plane is related to all threading dislocations (TDs), including pure edge, screw and mixed dislocations^31,32^. The FWHM values of both of the (002) peak and (102) peak for the HVPE-GaN crystals grown on the ECMGA and TEMGA templates are lower than that from the MGA template. It means that both of the TDs density of the HVPE-GaN crystals grown on the TEMGA and ECMGA templates were lower. This result suggests that the utilization of the nanoporous template could improve the crystalline quality of HVPE-GaN crystals. The FWHM value of the (102) peak for the HVPE-GaN crystal grown on the TEMGA template is similar to that from the ECMGA template, while the FWHM value of the (002) peak for the HVPE-GaN crystal grown on the TEMGA template is lower than that from the ECMGA template. It means that the density of the screw or mixed dislocations of the HVPE-GaN crystals grown on the TEMGA template is lower than that from the ECMGA template. This can be explained by dislocation selective etching of the wet chemical etching process. According to Cabrera’s thermodynamic theory, the wet chemical etching is selective for the dislocation because of the energy localized in the vicinity of a dislocation. The screw and mixed dislocations are more active to wet chemical etching than the edge dislocations due to the lower free energy change^23^. Thus, the density of mixed and screw dislocations in TEMGA template is decreased much more than that of the edge dislocation after the wet chemical etching. Consequently, the density of mixed and screw dislocation in GaN crystal is decreased much more than the edge dislocation. Then, the FWHM value of (002) peak for the HVPE-GaN crystal grown on the TEMGA template is reduced. This result suggests that the utilization of the TEMGA template could improve the crystalline quality of HVPE grown GaN crystals.

**Figure 5:**
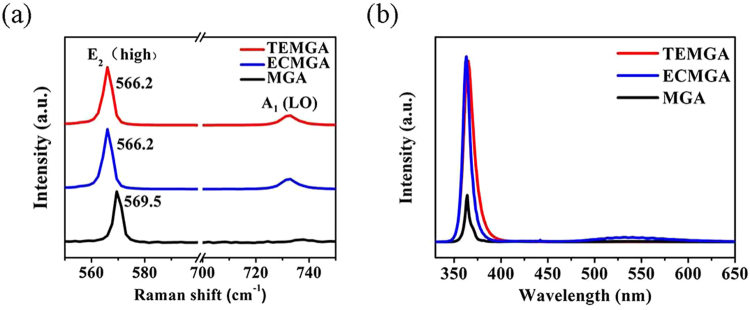
Raman spectra (**a**) and PL spectra (**b**) of the HVPE grown GaN crystals with TEMGA, ECMGA and MGA template.

The Raman spectrum was used to research the residual stress of the HVPE-GaN crystals grown on different substrates. Figure 5(a) shows the Raman spectra of the HVPE-GaN crystals grown on the TEMGA, ECMGA and MGA templates. The E_2_ (high) phonon mode is believed to be only related to the biaxial stress in the crystal. The stress can be calculated by the following equation^33^:

1 $$\sigma =\frac{{\rm{\Delta }}\omega }{4.3}({\rm{cm}}\,GPa)$$

where σ is the biaxial stress and the Δω is the E_2_ (high) phonon peak shift. It is reported that E_2_ (high) mode phonon peak of the stress-free GaN is 566.2 cm^−1^ ^34^. The E_2_ (high) mode phonon of the GaN grown on the TEMGA and MGA templates are 566.2 cm^−1^ and 569.5 cm^−1^, respectively. Then, the compressive stress of the GaN grown on the TEMGA and MGA templates are calculated as 0 and 0.76 GPa which indicates that the residual stress of HVPE-GaN crystals grown on TEMGA template was greatly reduced. The voids formed from the nanopores after the HVPE growth played a key role in the stress release process.

The optical quality of the HVPE-GaN crystals grown on the TEMGA, ECMGA and MGA templates was characterized by room temperature PL spectroscopy (Fig. 5(b)). Strong band edge emission peaks is observed at 365 nm. TDs existing in GaN crystal play the role of non-radiative recombination centers to deteriorate the luminescence efficiency. Therefore, the band edge emission intensity of GaN grown on TEMGA template is almost equal to that of GaN grown on ECMGA template, which is thrice higher than that of GaN grown on MGA template. A weak yellow luminescence band which is related to point defects such as Ga vacancy and impurity^35^ is observed at 500–600 nm. The intensity of the yellow luminescence peaks of HVPE-GaN crystals grown on MGA, ECMGA and TEMGA is low. These results indicate that the crystalline quality of GaN grown on TEMGA template was improved.

**Figure 6:**
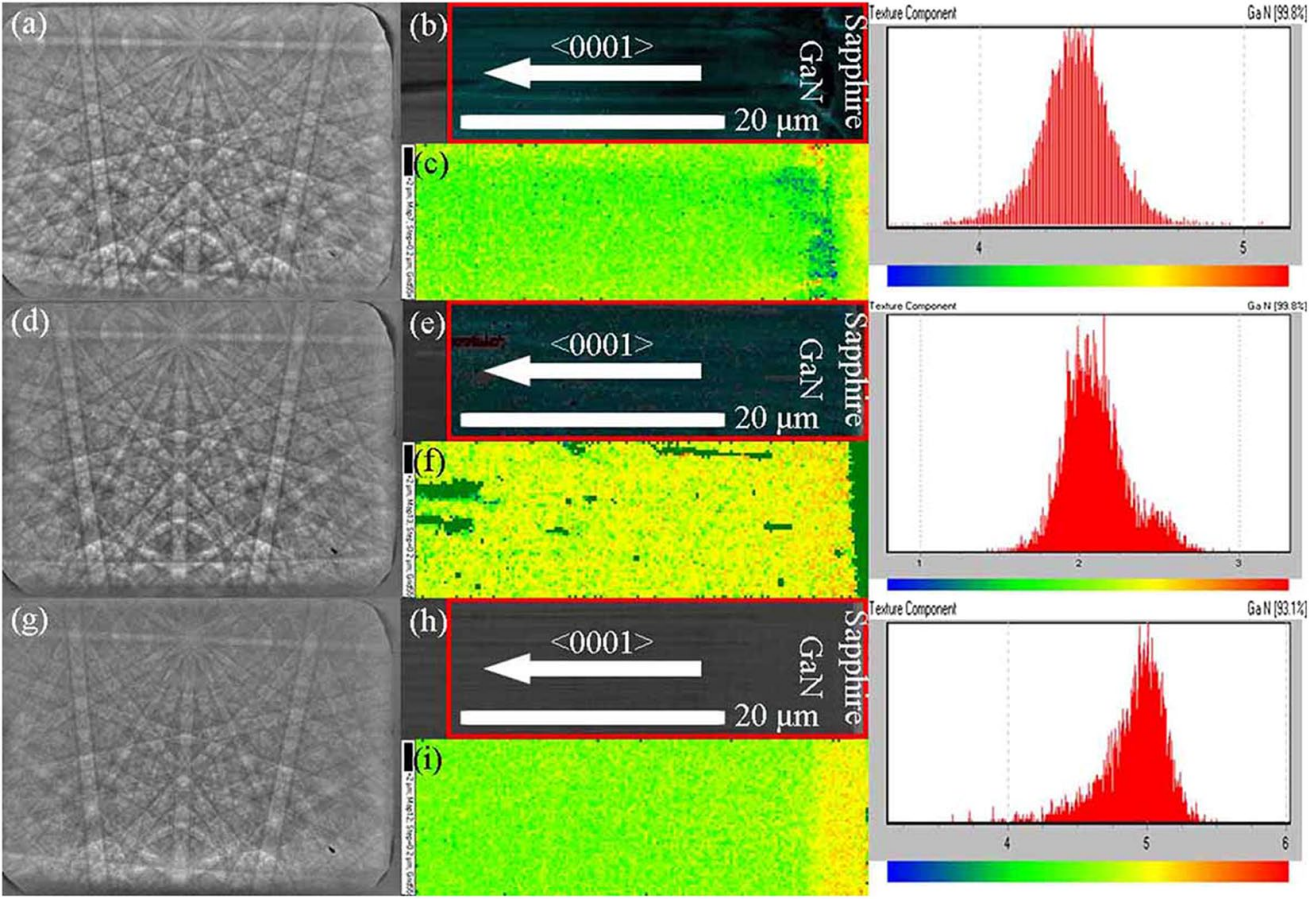
EBSD Kikuchi patterns (**a**), SEM image of mapping area (**b**) and texture component result (**c**) of GaN crystals grown on TEMGA template. EBSD Kikuchi patterns (**d**), SEM image of mapping area (**e**) and texture component result (**f**) of GaN crystals grown on ECMGA template. EBSD Kikuchi patterns (**g**), SEM image of mapping area (**h**) and texture component result (**i**) of GaN crystals grown on MGA template.

Electron backscatter diffraction (EBSD) is an effective method for studying the crystal phase, orientation, and lattice strain variations in the crystal materials^36–38^. Figure 6(a,d and g) show the EBSD Kikuchi patterns of HVPE-GaN crystals grown on TEMGA, ECMGA and MGA templates, respectively. The high-quality diffraction pattern provides the crystallographic orientation information of HVPE-GaN crystals grown on the TEMGA template. Figure 6(b,e and h) show the mapping area SEM image of HVPE-GaN crystals grown on TEMGA, ECMGA and MGA templates, respectively. Figure 6(c,f and i) show the texture component results obtained from the EBSD mapping data of mapping area in Fig. 6(b,e and h), respectively. In Fig. 6(c), it is observed that the mapping color near the interface is yellow, the mapping color of the nanoporous buffer layer is a mixture of green and blue and the mapping color of the epitaxial GaN is green. These results indicate that the disorientation near the interface is the largest and the disorientation of the nanoporous buffer layer is the smallest in GaN grown on TEMGA template. The strain was in-direct proportion to the lattice deformation. Therefore, the strain of the nanoporous buffer layer is the smallest in GaN grown on TEMGA template. In Fig. 6(f), the mapping color is a mixture of green and yellow all the map, and the density of the yellow dots near the interface is higher than that in epitaxial GaN. Thus, the disorientation near the interface is also the largest, but the difference is very small in this sample. These results indicate that the stain almost remain constant in GaN grown on ECMGA. In Fig. 6(i), the mapping color near the interface is a mixture of yellow and red and the mapping color of epitaxial GaN is green which means that the disorientation near the interface is much larger than that far from it and the strain near the interface is the largest. The EBSD mapping data of HVPE-GaN crystals grown on TEMGA, ECMGA and MGA templates intuitively show that the nanoporous layer of TEMGA and ECMGA templates can release the strain caused by the lattice and thermal expansion coefficient mismatches at the interface.

## Conclusion

In conclusion, we investigated the structure of the porous layer obtained by the two-step etching method. The as-obtained porous layer with high porosity and rather smooth surface exhibited advantages of porous structure obtained by the electrochemical and the H_3_PO_4_ etching. High porosity was essential to the formation of well-aligned voids that assisted the self-separating of HVPE grown GaN crystal from the substrate. The porous structure played a significant role in the stress release and self-separating process. The good optical quality of GaN grown on the TEMGA template was demonstrated by PL spectra. The EBSD confirmed that the residual stress was released through the porous layer. These results showed that the two-step etching method was an effective approach for the growth of the free-standing GaN crystals. The two-step etching method could also be utilized for fabricating GaN-based devices.
